# Selective facilitation of short latency postural reflexes by instability

**DOI:** 10.1007/s00221-025-07168-8

**Published:** 2025-10-04

**Authors:** Sendhil Govender, Daniel Hochstrasser, Neil P. M. Todd, James G. Colebatch

**Affiliations:** 1 Neuroscience Research Australia, University of New South Wales, Randwick, Sydney, NSW 2052 Australia; 2 School of Clinical Medicine, Randwick Clinical Campus, University of New South Wales, Randwick, Sydney, NSW 2052 Australia; 3 MARCS Institute for Brain, Behaviour and Development, Western Sydney University, Westmead, Sydney, NSW 2145 Australia; 4 Institute of Neurological Sciences, Prince of Wales Hospital, Randwick, Sydney, NSW 2031 Australia

**Keywords:** Postural reflexes, Instability, Perturbation, Balance

## Abstract

We sought to determine if enhancement of postural reflexes occurs specifically in response to instability induced by leaning and the possible neural level at which this may be mediated. Healthy participants (*n* = 10; 37 ± 15 years) were asked to adopt leaning postures to increase postural instability. Recordings of postural, eye and neck muscles and from over the cerebellum were made during stable upright stance and with the feet plantarflexed or dorsiflexed. The latter postures were used to modulate tonic activity levels in the soleus and tibialis anterior (TA) muscles to a similar degree to leaning. External perturbations were delivered to the trunk [vertebra prominens (C7) and upper sternum] and at the mastoids. C7 stimulation produced larger short latency (SL) reflex amplitudes in soleus during forwards lean than upright stance, when using matched tonic levels, although SL amplitudes in TA in response to sternal stimulation were unaffected when tonic activity was matched. Cerebellar evoked responses were larger during anterior than posterior lean for truncal stimuli. Mastoid stimulation generally evoked larger responses in the legs during lean than during upright stance with matching tonic EMG levels. Vestibular and non-vestibular responses recorded from the sternocleidomastoid muscles were often larger during posterior lean, independent of the direction of mastoid stimulation. Short-latency vestibular evoked cerebellar responses and extraocular responses were unaffected by posture. Facilitation of postural reflexes by instability occurred for axially-evoked postural reflexes in soleus but not TA. We propose that this effect is mediated by modulation of descending brainstem projections.

**Supplementary Information:**

The online version contains supplementary material available at 10.1007/s00221-025-07168-8.

## Introduction

Postural reflexes maintain balance and upright stance in response to unexpected perturbations. Sudden disturbances applied at the ankle or to the upper trunk during standing evoke well-defined, short-latency lower limb EMG responses. These occur at ~ 46 ms for tap stimuli (Bötzel et al. 2001), 55–65 ms for platform displacements (Diener et al. 1984), and 56–58 ms for axial perturbations (Govender et al. 2015). Comparable latencies have been reported following vestibular activation: 55–65 ms with galvanic stimulation (Britton et al. 1993; Fitzpatrick and Day 2004) and ~ 54 ms for impulsive mastoid accelerations (Laube et al. 2012). These are well below voluntary reaction times for the legs (Thompson et al. 1992).

Postural control is enhanced with greater postural threat, such as standing at an elevation (Adkin et al. 2000). When upright stance is unstable, galvanic (vestibular) stimulation produces larger leg EMG responses (Fitzpatrick et al. 1994) and increased sway (Horak and Hlavacka 2001). Leaning forwards or backwards acts to destabilise posture and increases tonic activity in leg muscles. Similar postures are commonly adopted when postural reflexes are examined (Britton et al. 1993; Welgampola and Colebatch 2001). Perturbation-evoked leg EMG responses become larger as subjects lean and the potential threat to stability increases but this also increases tonic activity in the target muscles (Govender et al. 2015). Tonic activation is expected to increase reflex amplitudes approximately linearly when, as is usually the case, afferents project throughout the entire motoneurone pool (Matthews 1986). A clear example of the dependency of reflex amplitude on tonic EMG activity is the cervical vestibular evoked myogenic potential (cVEMP; Colebatch et al. 1994; Rosengren 2015). Deviations from linearity may occur. For example, phenomena such as plateau potentials (e.g. Heckman et al. 2008; Kudina and Andreeva 2010) would not be expected to scale linearly. These considerations emphasise that matching the tonic levels of contraction should be done when comparing reflex gains.

Bötzel et al. (2001) found that brief mechanical taps to the sternum evoked short-latency tibialis anterior (TA) responses that were posture-dependent and independent of vestibular input, with larger responses during posterior lean than neutral stance. Govender et al. (2015) showed that controlled axial perturbations at the trunk produced direction and posture-specific short-latency responses: stimulation at the vertebra prominens (C7) elicited larger soleus responses during anterior lean, whereas sternal stimulation evoked larger TA responses during posterior lean. In both cases, increased reflex amplitudes were accompanied by greater tonic activity in the target muscles, making it unclear whether the enhancement reflected elevated tonic muscle activity or the added instability of leaning. The effects of perceived postural instability and threat may extend beyond postural reflexes. Height-induced postural threat has been reported to modulate vestibular responses from the sternocleidomastoid (SCM) and extraocular muscles (Naranjo et al. 2015, 2016).

In this study, we sought to determine whether postural reflexes are specifically enhanced by instability induced through leaning, while controlling for differences in tonic muscle activity. We have previously reported the characteristics of evoked postural responses to brief pulses of acceleration applied to the upper trunk (i.e. miniperturbations) (Govender et al. 2015, 2022; Graus et al. 2013) and to the mastoids (Laube et al. 2012; Govender et al. 2024a). The axial perturbations do not depend upon vestibular afferents whereas the mastoid taps do (Laube et al. 2012; Graus et al. 2013). Both these forms of stimulation evoke short latency (SL) reflex responses in lower limb muscles (onset at 53–59 ms), appropriate for compensating for the applied disturbance. In order to determine whether enhancement occurs specifically due to instability we matched the tonic muscle activation levels between the leaning and upright postural conditions. We also recorded evoked potentials from over the cerebellum, SCM muscles and beneath the eyes to determine how widespread any effects might be and thus also potentially deduce the level of the neuraxis at which any effect operated. For mastoid stimulation, ocular and cervical vestibular evoked myogenic potentials (oVEMPs and cVEMPs) were recorded, the former bilaterally (n12, n15) and the latter in the SCM contralateral to the direction of head rotation (p15, p20), along with vestibular cerebellar evoked potentials (VsCEPs; Govender et al. 2022; Todd et al. 2018).

## Materials and methods

### Participants

Ten healthy participants (37 ± 15 years; 6 males, 4 females) from staff and students at the Prince of Wales Hospital, Western Sydney University and the general community were tested. Participants reported no prior history of inner ear pathology or neurological deficits. A subset had acceleration and force platform measurements (48 ± 16 years; 3 males, 2 females). The attrition rate was zero. Informed consent was obtained prior to the study commencing. The study was approved by the South Eastern Sydney Local Health District Human Research Ethics Committee and conducted in accordance with the 1964 Declaration of Helsinki and its subsequent amendments.

### Perturbation stimuli

The stimuli consisted of small perturbations which were produced using a brief impulsive waveform (a 3rd order gamma waveform with a 4 ms rise time; for illustrations see Todd et al. 2008; Graus et al. 2013; Govender et al. 2024b). Impulses were generated using a laboratory interface (CED Power1401, Cambridge Electronic Design, Cambridge, UK), a power amplifier (model 2718, Brűel & Kjær, Denmark) and customised software. The stimulus was delivered by a single experimenter using a hand-held mini-shaker device (model 4810, Brüel & Kjaer P/L, Denmark) with an attached cylindrical perspex rod (diameter: 2.5 cm, length 9.2 cm). The mini-shaker was applied to the upper sternum, the spinous process of the vertebra prominens and the mastoids separately with a constant pressure. The initial displacement of the rod was towards the subject (positive polarity) at a fixed intensity of 20 Volts peak (~ 14 N peak force level (FL) and induced accelerations of the head and trunk of around 50 mg (Todd et al. 2008; Govender et al. 2024a, b). Stimuli were applied at a rate of ~ 2 Hz and each recording consisted of 60–120 individual trials. All recordings were sampled at 4096 Hz with an epoch of 400 ms (200 ms before and after stimulus onset).

### EEG, extraocular and EMG recordings

EEG was recorded using a custom 10–10 cerebellar-extended cap (EASYCAP GmbH, Germany). A subset of electrodes was used based on our previous work (Govender et al. 2020, 2022) and consisted of the Iz, PO9 and PO10 locations. The ground electrode was positioned at Cz and a reference electrode at AFz. Extraocular signals were recorded with active electrodes beneath each eye on the infraorbital margin and reference electrodes directly below. EEG and extraocular signals were amplified (20,000×) and filtered (0.5 Hz to 3 kHz) using D360 amplifiers (Digitimer Ltd, Welwyn Garden City, U.K.).

Unrectified and rectified EMG was recorded bilaterally using self-adhesive electrodes (Cleartrace 1700-030, Conmed Corp., NY, USA) from the SCM, TA and soleus muscles using a bipolar montage. For the neck muscles, active electrodes were placed just above the midpoint of the SCM with reference electrodes over the sternoclavicular joints (Rosengren et al. 2019). For the lower limbs, active electrodes were positioned 1–2 cm distal to the gastrocnemius musculotendinous junction for soleus and 1–2 cm lateral to the anterior border of the tibia for TA. Reference electrodes were placed 2 cm below the active electrodes (center-to-center electrode distance). A ground electrode was placed on the midpoint of the left lower leg on the medial (bony) surface of the tibia. EMG signals were amplified (2500x) and filtered (8 Hz to 1.6 kHz) using AA6 Mk III amplifiers (Medelec Ltd, Old Woking, Surrey UK) and recorded using Signal software and a Power1401 (Cambridge Electronic Design, Cambridge, UK).

### Acceleration and force plate recordings

Head accelerations were measured using a triaxial piezoelectric accelerometer (model 2228 C-Y, Endevco) positioned over the nasion. Truncal accelerations were recorded with a uniaxial accelerometer (model 751-100, Endevco) placed at C7 (during sternal and mastoid stimulation) or at the sternum (during C7 stimulation). Centre of pressure (CoP) displacement was measured using a force platform (model 9286 A, Kistler Instrumente, Winterthur, Switzerland).

### Experimental procedure

Twenty recordings were made using three different stimulus sites (the sternum, C7 spinous process and mastoids). Subjects were given a rest period of 1–2 min between each recording. All recordings were made during standing with feet together. For all stimulation sites, subjects were instructed to maintain one of three standing postures: anterior lean, posterior lean or upright neutral stance. Participants were instructed to lean forward or backward to the maximum extent they felt was safe. Their eyes were open throughout with gaze directed upwards to facilitate oVEMP responses (Govender et al. 2009). Additional recordings were made for upright neutral stance during induced plantarflexion and dorsiflexion of the feet, using a wooden block inserted under the toes or heels. This allowed for tonic EMG activation levels to be closely matched during the lean and upright neutral stance conditions for the agonist muscle group. Monitoring of rectified EMG levels was available in real-time using signal software (Cambridge Electronic Design, Cambridge, UK). For mastoid stimulation the subject’s head was turned as far as comfortably possible to either the left (*n* = 5 subjects) or right (*n* = 5) sides with both mastoids tested for each standing posture. The order of conditions was randomised. Acceleration and force platform recordings were obtained separately across all twenty recording conditions in a subset of five subjects.

### Measurements

For the lower limbs, rectified EMG averages from the soleus and TA muscles were used to measure baseline levels, SL amplitudes and SL latencies. Baseline (tonic) levels were measured over the prestimulus interval (200 ms before onset). SL amplitudes were measured using the average level of rectified EMG during the period of excitation or inhibition and expressed as a percentage of baseline EMG levels (Govender et al. 2015; Graus et al. 2013). SL latencies were measured from the time of stimulation to the onset of the SL response (SL onset) and from time to stimulation to the end of the SL response (SL end).

For SCM and extraocular potentials, amplitudes and latencies were measured from unrectified EMG averages. Stretch reflex responses in the SCM were measured as biphasic (n35, p49; C7 stimulation) and triphasic (p25, n37, p50; sternal stimulation) waveforms (Todd et al. 2022). During mastoid stimulation, responses were measured for the cVEMP (p15 and p20 peaks) and the later stretch reflex response (n2 peak). For the extraocular muscles, the n26 peak was measured for C7 stimulation, n21 peak for sternal stimulation and n12/15 peaks for mastoid stimulation. The SCM and ocular evoked responses were labelled ipsilateral or contralateral to the direction of head rotation for mastoid stimulation (Laube et al. 2012).

For EEG measurements, peak amplitudes and latencies were based on our previous observations at the Iz electrode as an indicator of cerebellar activation (Govender et al. 2022). The Iz response was measured as P25 and P50 peaks for C7 stimulation (Govender et al. 2022; Todd et al. 2021) while the P23 and P54 peaks were measured for sternal stimulation (Govender et al. 2024b). For mastoid stimulation, vestibular evoked cerebellar responses were measured from the contralateral PO9 or PO10 electrodes relative to the direction of head rotation (Govender et al. 2020; Todd et al. 2018).

For head accelerations, amplitude and latency were measured at the initial peak along the naso-occipital (x), vertical (z), and interaural (y) axes. For trunk accelerations, amplitude and latency measurements were taken from the dominant initial peak. CoP displacement was calculated in the anteroposterior (AP) plane using custom MATLAB software (Mathworks, MA, USA). Peak CoP measurements were used to determine displacement after stimulus onset.

### Statistical analysis

Statistical analysis was conducted using SPSS (version 27.0, IBM Inc., Chicago, USA). The mastoid on the side opposite to the rotated head was labelled as the anterior mastoid and the mastoid on the same side as the rotated head labelled as the posterior mastoid. Responses from the right and left sides (or ipsilateral and contralateral sides for mastoid stimulation) were averaged when tonic levels, amplitudes or latencies were not significantly different between them. Paired sample *t* tests were used to compare tonic levels, SL amplitudes and SL latencies separately between anterior lean and neutral stance with plantarflexion conditions (C7 and posterior mastoid stimulation) and between posterior lean and neutral stance with dorsiflexion (sternal and anterior mastoid stimulation).

One way repeated measures analysis of variance (ANOVA) using posture (3 levels; anterior, neutral and posterior) as the factor was used to compare Iz, SCM and extraocular responses separately. Repeated measures ANOVA comparing responses across the neutral stances (3 levels; neutral, neutral with dorsiflexion and neutral with plantarflexion) were also carried out. Accelerations amplitudes and latencies were analysed using posture as the factor (with 5 levels: anterior, neutral, neutral with dorsiflexion, neutral with plantarflexion and posterior). The Greenhouse–Geisser correction was used to correct for violation of the assumption of sphericity. Post-hoc paired *t* tests were used when significant main effects were found on ANOVA and are reported in the text. The level of significance was set at *p* < 0.05 with trends identified when *p* < 0.1. Effect sizes are reported using Cohen’s *d* for *t* tests and partial eta squared (η_p_^2^) for ANOVAs. Values are given as mean ± SD in tables and mean ± SEM in graphs.

## Results

### C7 stimulation

**Fig. 1:**
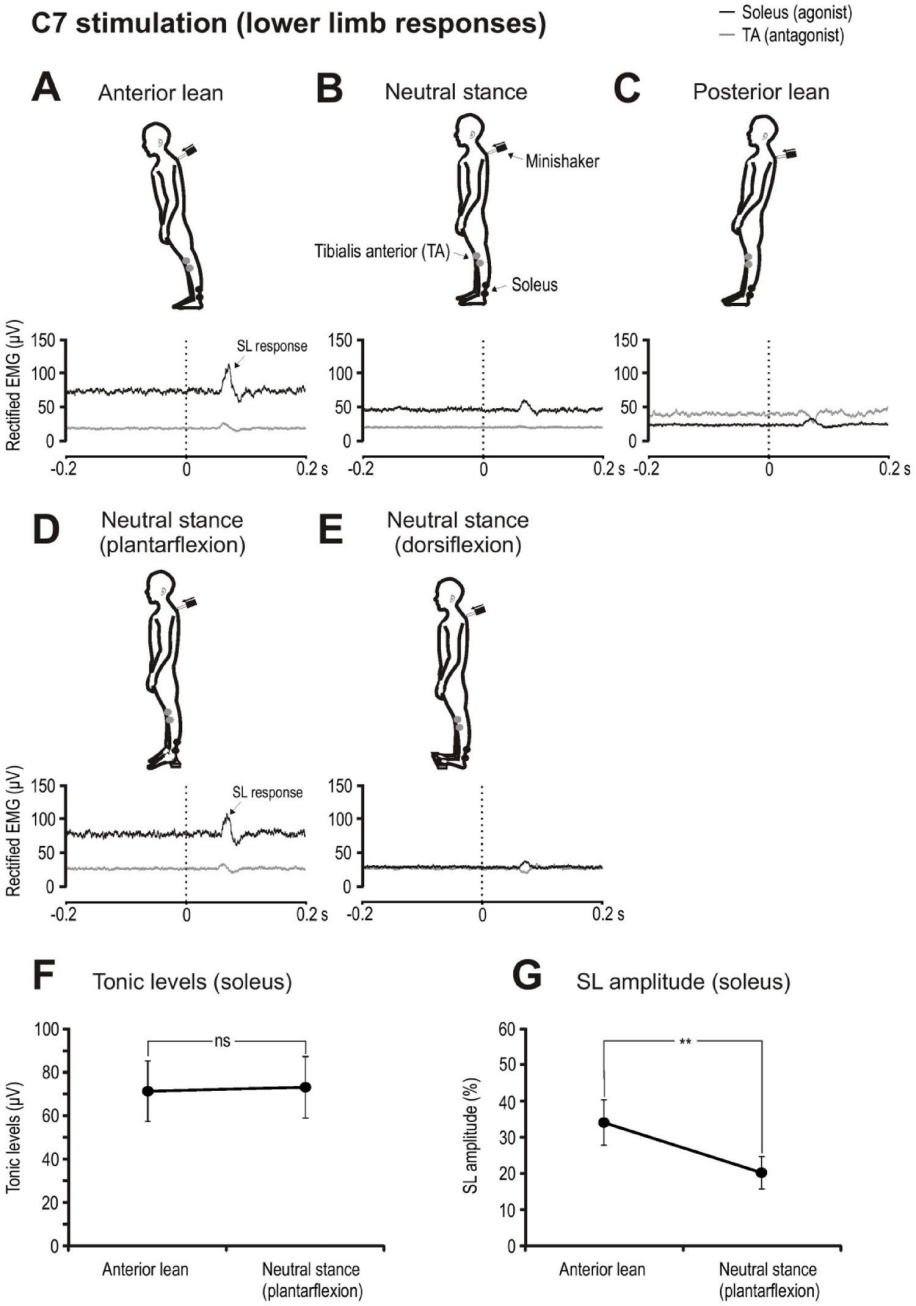
Grand mean EMG evoked responses to C7 stimulation during leaning and upright postures. Anterior lean produced larger tonic levels and SL responses in the agonist muscle (soleus; black traces) (**A**–**C**). Plantarflexion during upright stance increased tonic levels in soleus (**D**). Dorsiflexion increased tonic levels in the antagonist (TA) muscle resulting in similar levels to that of soleus (**E**). Tonic soleus EMG levels during anterior lean and plantarflexion postural conditions were similar (**F**), and the SL response was larger overall during the anterior lean condition (**G**). Traces in panels **A**–**E** show right sided EMG responses, but symmetrical responses were present. ns = not significant, **p < 0.01. Vertical dashed lines reflect stimulus onset in all figures

For stimulation applied at C7, the imposed transient is resisted by the action of soleus. The stimulus produced SL (mean onset latency approximately 60 ms post stimulus) excitatory responses in the soleus muscles which became larger with anterior lean (Fig. 1A–C). Tonic levels during anterior lean (71.4 ± 44.0 µV) were greater than levels recorded during neutral stance (43.0 ± 28.1 µV) and posterior lean (21.9 ± 7.9 µV). Neutral stance with plantarflexion increased tonic activity in soleus (Fig. 1D) to closely match that for anterior lean (Table 1; Fig. 1F). Despite similar levels of tonic activation, SL amplitudes were significantly larger during anterior lean than when standing upright with the soleus muscles plantarflexed (34 ± 20% vs. 20 ± 14%; t_(9)_ = 3.5, *p* = 0.006, *d* = 1.1, Fig. 1G). Eight of ten subjects had larger SL responses during anterior lean compared to upright stance with plantarflexion. SL onset and end times did not differ between anterior lean and the plantarflexed conditions (Table 1).

For the antagonist muscle (TA), tonic activity increased with leaning backwards and with dorsiflexion when standing upright. With higher levels of activation, SL inhibition became evident in the TA muscle (Fig. 1C, E).

**Table 1:** EMG tonic levels, amplitudes and latencies recorded from soleus during C7 stimulation and TA during sternal stimulation (*n* = 10)

C7 stimulation			
	Anterior lean	Neutral stance with plantarflexion	*p* value
	Soleus	Soleus	
Tonic levels (µV)	71.4 ± 44.0	73.2 ± 44.8	0.778
SL amplitude (%)	34 ± 20	20 ± 14	0.006**
SL onset (ms)	57.2 ± 4.3	58.8 ± 5.4	0.273
SL end (ms)	78.2 ± 6.0	75.5 ± 7.2	0.146

Sternal stimulation			
	Posterior lean	Neutral stance with dorsiflexion	*p* value
	TA	TA	
Tonic levels (µV)	65.7 ± 42.6	63.2 ± 49.4	0.908
SL amplitude (%)	55 ± 25	51 ± 36	0.698
SL onset (ms)	59.0 ± 10.5	57.3 ± 9.2	0.204
SL end (ms)	80.3 ± 5.6	80.3 ± 8.2	0.996

Mean (SD), % = percentage change from tonic levels. **p < 0.01

**Fig. 2:**
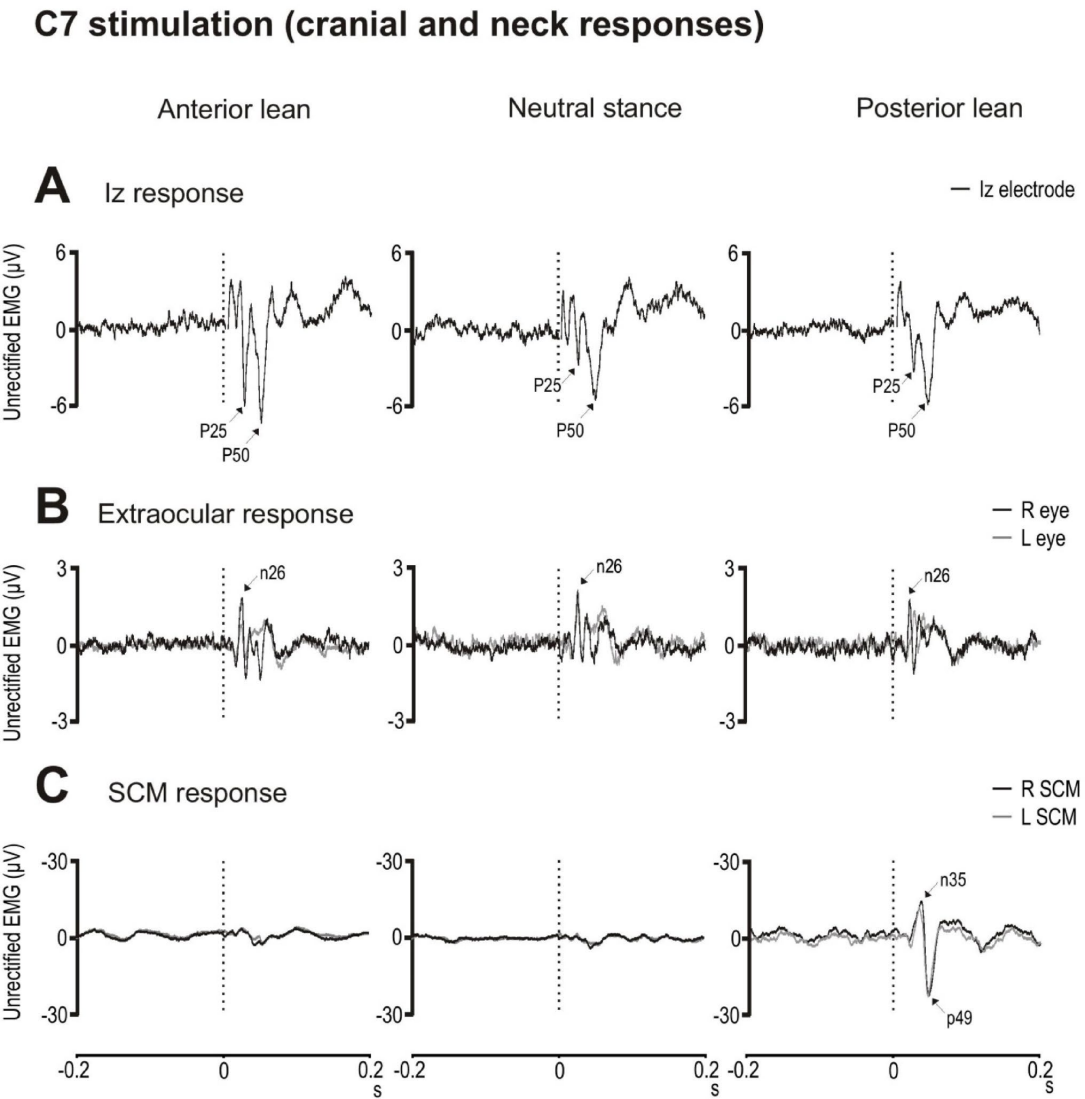
Grand mean evoked responses following C7 stimulation recorded from the Iz electrode (**A**), extraocular muscles (**B**) and sternocleidomastoid (SCM) muscles (**C**) during anterior lean (left column), neutral stance (middle column) and posterior lean (right column) postural conditions

At the Iz electrode, the response consisted of a series of waves with the P25 and P50 peaks being the most consistent and prominent (Fig. 2A). Posture modulated Iz response amplitudes (F_(2,18)_ = 5.8 & 6.4, *p* = 0.03 & 0.01, η_p_^2^ = 0.39 & 0.42), consistent with our previous observations (Govender et al. 2022), with the P25 and P50 peaks being larger during anterior and neutral lean compared to posterior lean (t_(9)_ = 2.4 to 4.3, *p* = 0.04 to 0.002, *d* = 0.8 to 1.4). P25 and P50 latencies were unaffected (Table 2). Neither the P25 nor the P50 amplitudes differed across the three neutral stance conditions (F_(2,18)_ = 0.4 & 1.0, *p* = 0.693 & 0.389). Latencies for the P25 and P50 were also unaffected across the neutral stance conditions (F_(2,18)_ = 0.3 & 0.7, *p* = 0.781 & 0.525).

For the extraocular responses, waveforms were similar across the postural conditions (Fig. 2B). There was no effect of leaning posture on either n26 peak amplitudes or latencies (Table 2). Amplitudes and latencies for the n26 peak were unaffected across the three neutral stance conditions (F_(2,18)_ = 0.03 & 0.5, *p* = 0.975 & 0.601).

For the SCM muscles, a negative–positive (n35–p49) reflex response was observed only during posterior lean (Fig. 2C) despite tonic levels in SCM being only slightly higher during posterior lean than anterior and neutral stance (Table 2).

**Table 2:** Amplitudes and latencies recorded from the Iz electrode and extraocular sites during C7 stimulation and tonic levels from SCM (*n* = 10)

C7 stimulation				
	Anterior lean	Neutral stance	Posterior lean	*p* value
Iz response				
P25 amplitude (µV)	7.9 ± 4.4	5.5 ± 3.3	3.7 ± 2.7	0.03*
P50 amplitude (µV)	13.7 ± 5.5	12.1 ± 7.4	7.7 ± 4.4	0.01*
P25 latency (ms)	26.3 ± 2.7	27.2 ± 1.5	26.8 ± 2.4	0.576
P50 latency (ms)	51.3 ± 6.2	50.6 ± 6.8	48.2 ± 3.5	0.109
Extraocular response				
n26 amplitude (µV)	2.0 ± 0.8	2.1 ± 1.3	1.9 ± 1.4	0.906
n26 latency (ms)	25.8 ± 1.8	26.1 ± 1.8	26.8 ± 1.7	0.312
SCM				
Tonic levels (µV)	30.1 ± 11.8	28.9 ± 10.2	35.4 ± 13.8	0.057

Mean (SD), Greenhouse–Geisser corrected *P* values are given. *p < 0.05

### Sternal stimulation

**Fig. 3:**
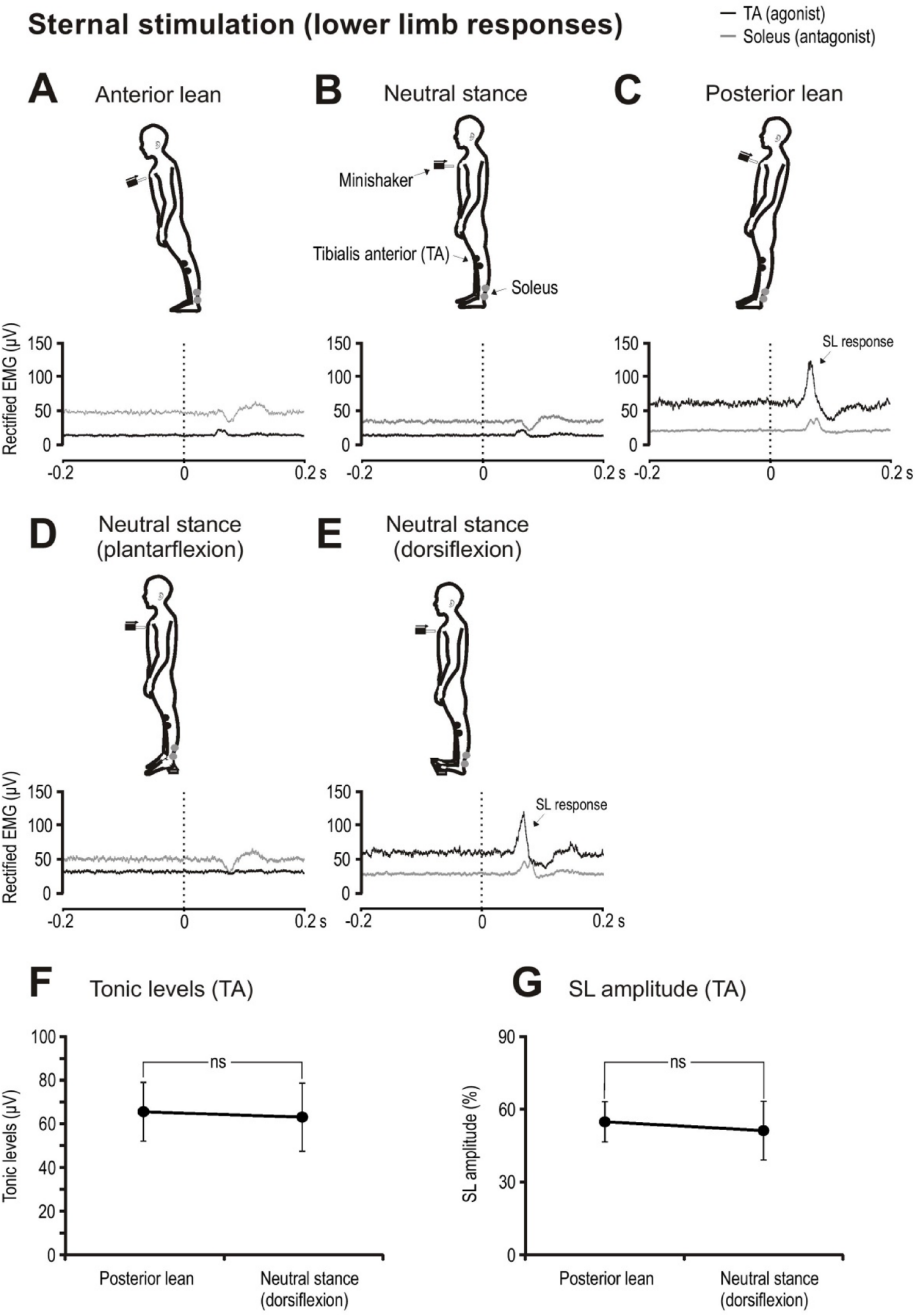
Grand mean EMG evoked responses to sternal stimulation during leaning and upright postures. Posterior lean produced larger tonic levels and SL responses in the agonist muscle (tibialis anterior (TA); black traces; **A**–**C**). Neutral stance conditions are shown with plantarflexion (**D**) and dorsiflexion (**E**) of the feet. Tonic levels (**F**) and SL amplitudes (**G**) were similar between the posterior lean and neutral stance with dorsiflexion conditions. Traces in panels **A**–**E** show right sided EMG responses. ns = not significant

For stimulation applied to the sternum, the imposed transient is resisted by the action of TA. Stimulation produced SL excitatory responses in the TA muscles which became larger with posterior lean (Fig. 3A–C). Tonic activity during posterior lean (65.7 ± 42.6 µV) was greater than levels during neutral stance (10.7 ± 7.5 µV) and anterior lean (12.6 ± 7.1 µV). Dorsiflexion during neutral stance increased tonic levels in TA (Fig. 3E) with the levels being closely matched to those of posterior lean (Table 1; Fig. 3F). With similar tonic activation levels for TA, there was no significant difference in SL amplitudes between posterior lean and dorsiflexed postural conditions (55 ± 25% vs. 51 ± 36%; t_(9)_ = 0.4, *p* = 0.698, Fig. 2G). Onset and end times also did not differ between posterior lean and dorsiflexed postural conditions (Table 1). Plantarflexion during neutral stance and during anterior lean attenuated the SL response in TA while in soleus (the antagonist) an SL inhibition became apparent (Fig. 3A, D).

**Fig. 4:**
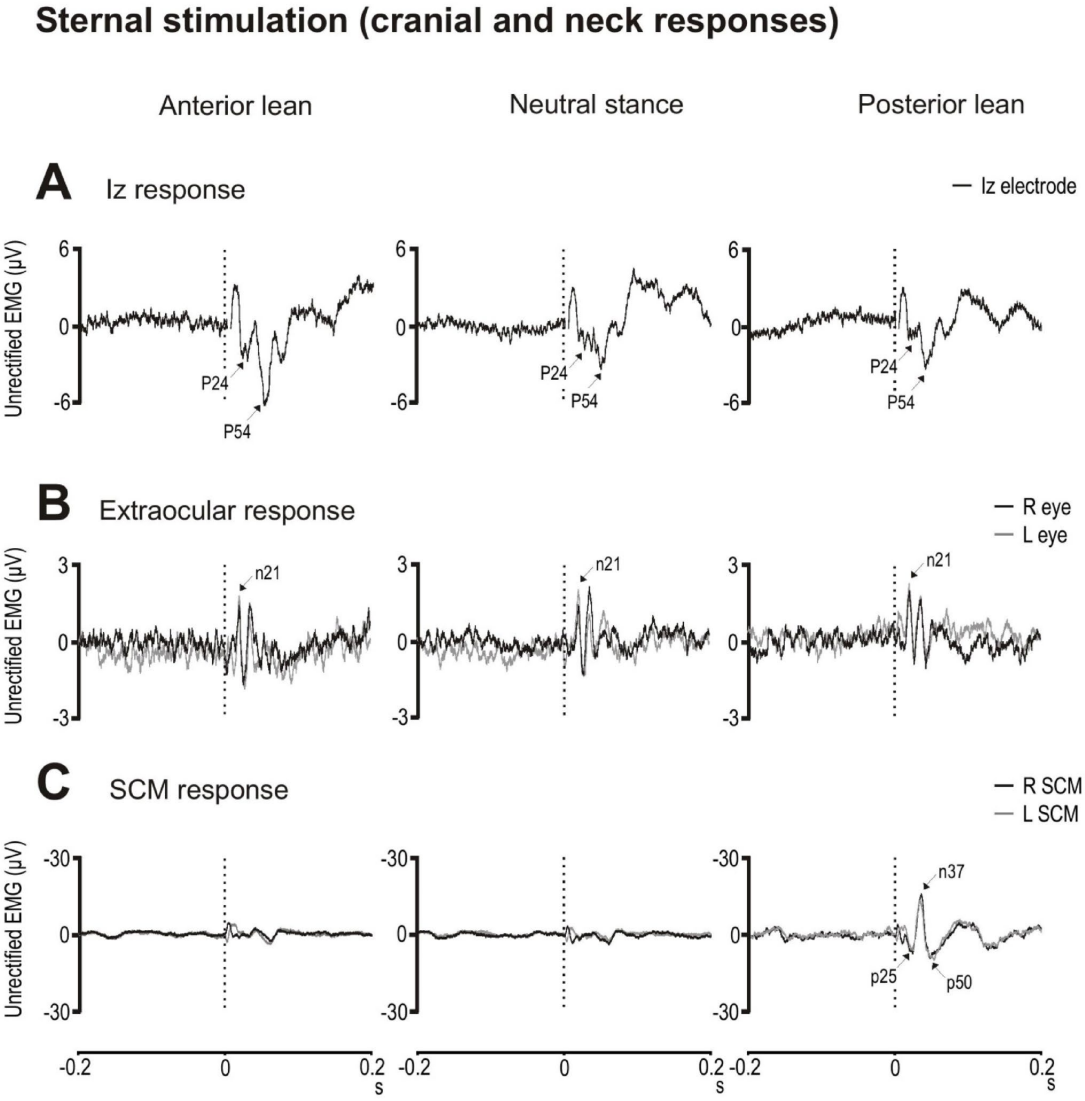
Grand mean evoked responses following sternal stimulation recorded from the Iz electrode (**A**), extraocular muscles (**B**) sternocleidomastoid (SCM) muscles (**C**) during anterior lean (left column), neutral stance (middle column) and posterior lean (right column) postural conditions

Sternal stimulation, like C7 stimulation, evoked a response at Iz consisting of two dominant positive potentials, the P24 and P54 peaks (Fig. 4A). Posture affected Iz response amplitudes (F_(2,18)_ = 4.3 & 10.0, *p* = 0.048 & 0.006, η_p_^2^ = 0.33 & 0.53; Table 3). For both the P24 and P54 peaks, amplitudes during anterior lean were significantly larger than during posterior lean (t_(9)_ = 4.0 & 3.4, *p* = 0.003 & 0.007, *d* = 1.3 & 1.1). Mean latencies ranged from 22.9 to 24.1 ms for the P24 peak and 48.5–53.1 ms for the P54 peak, but latencies did not differ significantly across the anterior, neutral and posterior postural conditions (Table 3). For the P24 and P54 peaks, amplitudes (F_(2,18)_ = 0.2 & 0.1, *p* = 0.823 & 0.899) and latencies (F_(2,18)_ = 0.4 & 1.1, *p* = 0.700 & 0.342) were unaffected across the three neutral stance conditions.

Extraocular responses were very similar across postural conditions with sternal stimulation (Fig. 4B). There was no effect of posture on n21 peak amplitudes and latencies (Table 3). Amplitudes and latencies also did not differ across the three neutral stance conditions (F_(2,18)_ = 0.1 & 1.2, *p* = 0.931 & 0.314) .

For the SCM muscles, a triphasic positive–negative–positive (p25–n37–p50) reflex was observed bilaterally during posterior lean only, associated with higher tonic levels (F_(2,18)_ = 11.7, *p* = 0.006, η_p_^2^ = 0.57; Fig. 4C; Table 3).

**Table 3:** Amplitudes and latencies recorded from the Iz electrode and extraocular sites during sternal stimulation and tonic levels from SCM (*n* = 10)

Sternal stimulation				
	Anterior lean	Neutral stance	Posterior lean	*p* value
Iz response				
P24 amplitude (µV)	4.8 ± 2.7	3.0 ± 2.4	2.1 ± 2.9	0.048*
P54 amplitude (µV)	9.6 ± 4.9	5.7 ± 4.3	4.4 ± 3.7	0.006**
P24 latency (ms)	22.9 ± 2.6	24.1 ± 3.4	23.2 ± 2.0	0.345
P54 latency (ms)	51.3 ± 7.6	53.1 ± 3.1	48.5 ± 8.2	0.335
Extraocular response				
n21 amplitude (µV)	1.9 ± 0.7	2.0 ± 1.4	2.2 ± 2.2	0.847
n21 latency (ms)	21.4 ± 1.5	21.5 ± 1.5	21.5 ± 1.0	0.925
SCM				
Tonic levels (µV)	28.2 ± 10.4	28.1 ± 10.6	34.9 ± 11.4	0.006**

Mean (SD), Greenhouse–Geisser corrected *P* values are given. *p < 0.05, **p < 0.01

### Mastoid stimulation

Stimulation applied to the mastoids, unlike the midline stimuli used above, is thought to primarily activate vestibular afferents (Laube et al. 2012; Todd et al. 2008). Mastoid stimulation produced significantly smaller SL responses compared to the axial stimuli for both the TA (13 ± 7% vs. 55 ± 25%; t_(9)_ = 4.7, *p* = 0.002, *d* = 1.6) and soleus muscles (6 ± 6% vs. 34 ± 20%; t_(9)_ = 4.4, *p* = 0.002, *d* = 1.4).

#### Posterior mastoid stimulation

**Fig. 5:**
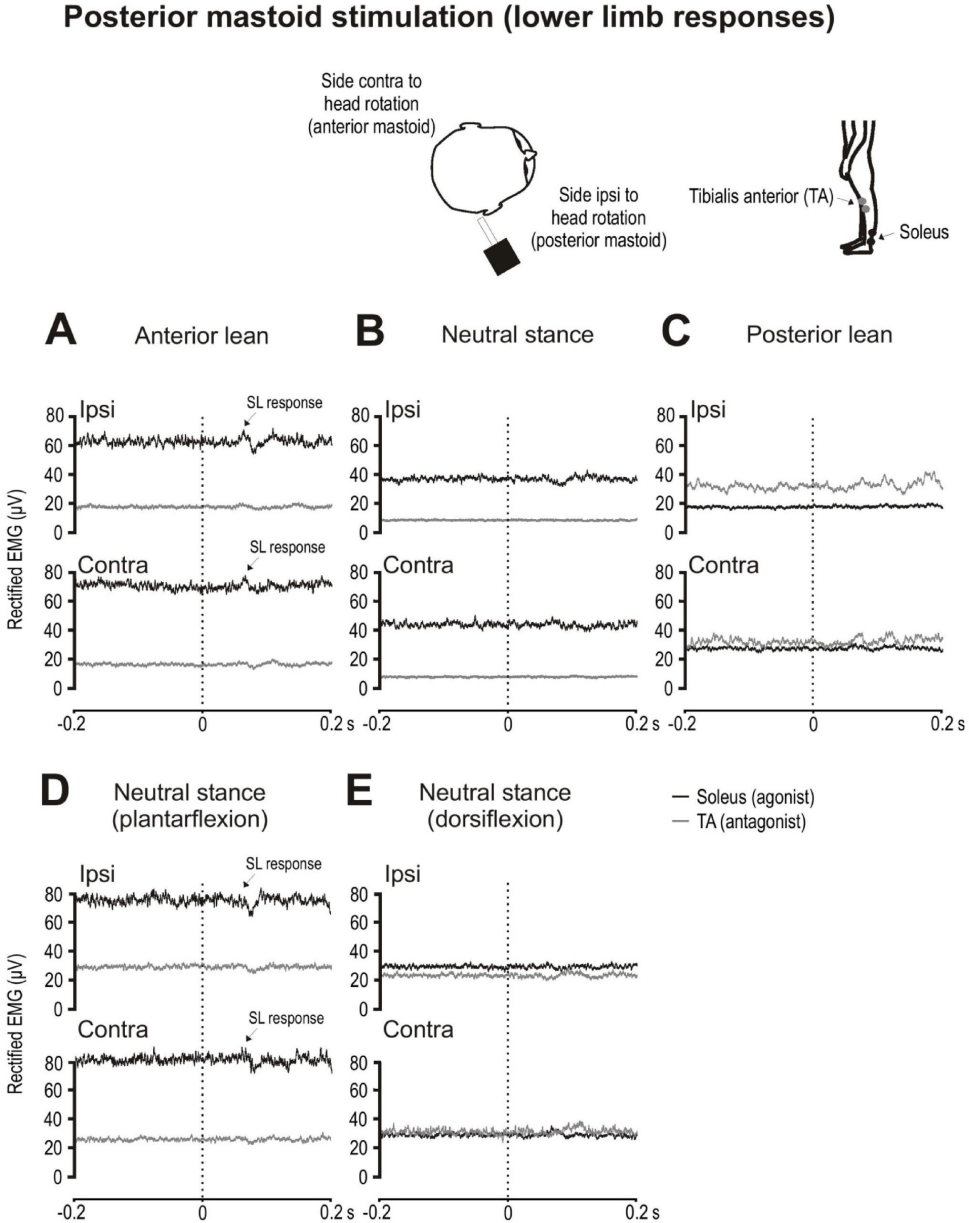
Grand mean EMG evoked responses to posterior mastoid stimulation during leaning and upright postures. Responses are shown ipsilateral and contralateral to the direction of head rotation Anterior lean increased tonic levels in soleus (black traces) and revealed excitatory SL responses bilaterally (**A**–**C**). SL responses could also be observed in soleus during neutral stance with plantarflexion (**D**). Little to no response was observed in both muscle groups with dorsiflexion (**E**)

Tonic levels for the soleus muscles were highest with anterior lean and for TA with posterior lean (Table 4). For posterior mastoid stimulation the anteriorly-directed perturbation was primarily resisted by soleus. Anterior lean produced excitatory SL responses in soleus (Fig. 5A). During upright neutral stance with plantarflexion (Fig. 5D) mean tonic levels for soleus showed a trend of being slightly higher than the anterior lean condition (79.0 ± 45.2 µV vs. 66.7 ± 49.6 µV t_(9)_ = 2.1, *p* = 0.069; Table 5). The soleus SL response showed a trend of being larger during anterior lean compared to the plantarflexed condition (6 ± 6% vs. 1 ± 8%; t_(9)_ = 2.1, *p* = 0.061). SL onset and end times did not differ between the anterior lean and the plantarflexed conditions (Table 5). There were no readily identifiable responses in either muscle during upright neutral stance with dorsiflexion (Fig. 5E).

**Table 4:** Tonic EMG, SCM, PO9/PO10 electrode and extraocular responses to posterior mastoid stimulation during anterior, neutral and posterior lean conditions (*n* = 10)

Posterior mastoid stimulation				
	Anterior lean	Neutral stance	Posterior lean	*p* value
Tonic EMG (lower limb muscles)				
TA (µV)	17.4 ± 10.6	8.5 ± 2.8	31.9 ± 35.4	0.056
Soleus (µV)	66.7 ± 49.6	40.6 ± 19.3	22.6 ± 8.2	0.003**
PO9/PO10 response (electrode contralateral to head rotation)				
P12 amp (µV)	63.4 ± 47.0	61.5 ± 41.5	62.1 ± 42.5	0.939
N17 amp (µV)	52.6 ± 50.7	47.4 ± 42.6	40.2 ± 36.4	0.432
P12–N17 pp amp (µV)	118.3 ± 93.3	110.7 ± 79.5	103.3 ± 74.7	0.664
P12 latency (ms)	11.1 ± 1.0	11.1 ± 1.0	11.5 ± 1.6	0.195
N17 latency (ms)	16.0 ± 2.2	15.8 ± 2.1	17.0 ± 4.2	0.335
Extraocular response				
n12 amp (µV)	3.2 ± 2.4	2.4 ± 1.9	4.2 ± 3.9	0.256
n15 amp (µV)	5.0 ± 3.0	3.9 ± 3.4	5.9 ± 5.1	0.270
n12 latency (ms)	13.0 ± 2.2	11.5 ± 1.1	12.3 ± 1.9	0.030*
n15 latency (ms)	16.5 ± 1.6	16.8 ± 1.6	17.3 ± 2.1	0.431
SCM response (SCM contralateral to head rotation)				
Tonic levels (µV)	33.9 ± 14.1	35.0 ± 13.5	47.5 ± 17.3	0.024*
p20 peak amp (µV)	10.8 ± 11.1	12.4 ± 11.8	24.4 ± 16.3	0.047*
p20 peak ratio	0.3 ± 0.3	0.3 ± 0.2	0.5 ± 0.2	0.056
n2 peak amp (µV)	20.3 ± 21.2	19.9 ± 23.7	47.6 ± 33.5	0.006**
n2 peak ratio	0.6 ± 0.4	0.5 ± 0.5	1.0 ± 0.5	0.001**
p20 latency (ms)	19.7 ± 3.3	21.4 ± 3.0	21.5 ± 2.0	0.118
n2 peak latency (ms)	37.8 ± 5.0	39.7 ± 3.4	36.0 ± 4.3	0.085

Mean (SD), amp = amplitude, pp = peak to peak, ratio = raw amplitude divided by tonic activity. Greenhouse–Geisser corrected *P* values are given. * *p* < 0.05, ** *p* < 0.01

**Table 5:** EMG tonic levels, amplitudes and latencies recorded from soleus during posterior mastoid stimulation and TA during anterior mastoid stimulation (*n* = 10)

Posterior mastoid stimulation			
	Anterior lean	Neutral stance with plantarflexion	*p* value
	Soleus	Soleus	
Tonic levels (µV)	66.7 ± 49.6	79.0 ± 45.2	0.069
SL amplitude (%)	6 ± 6	1 ± 8	0.061
SL onset (ms)	55.0 ± 8.4	54.4 ± 7.5	0.883
SL end (ms)	76.9 ± 8.8	72.5 ± 6.5	0.197

Anterior mastoid stimulation			
	Posterior lean	Neutral stance with dorsiflexion	*p* value
	TA	TA	
Tonic levels (µV)	48.5 ± 32.5	37.1 ± 33.8	0.433
SL amplitude (%)	13 ± 7	7 ± 6	0.087
SL onset (ms)	55.1 ± 7.0	50.8 ± 5.3	0.237
SL end (ms)	80.4 ± 8.8	81.7 ± 9.2	0.793

Mean (SD), % = percentage change from tonic levels

**Fig. 6:**
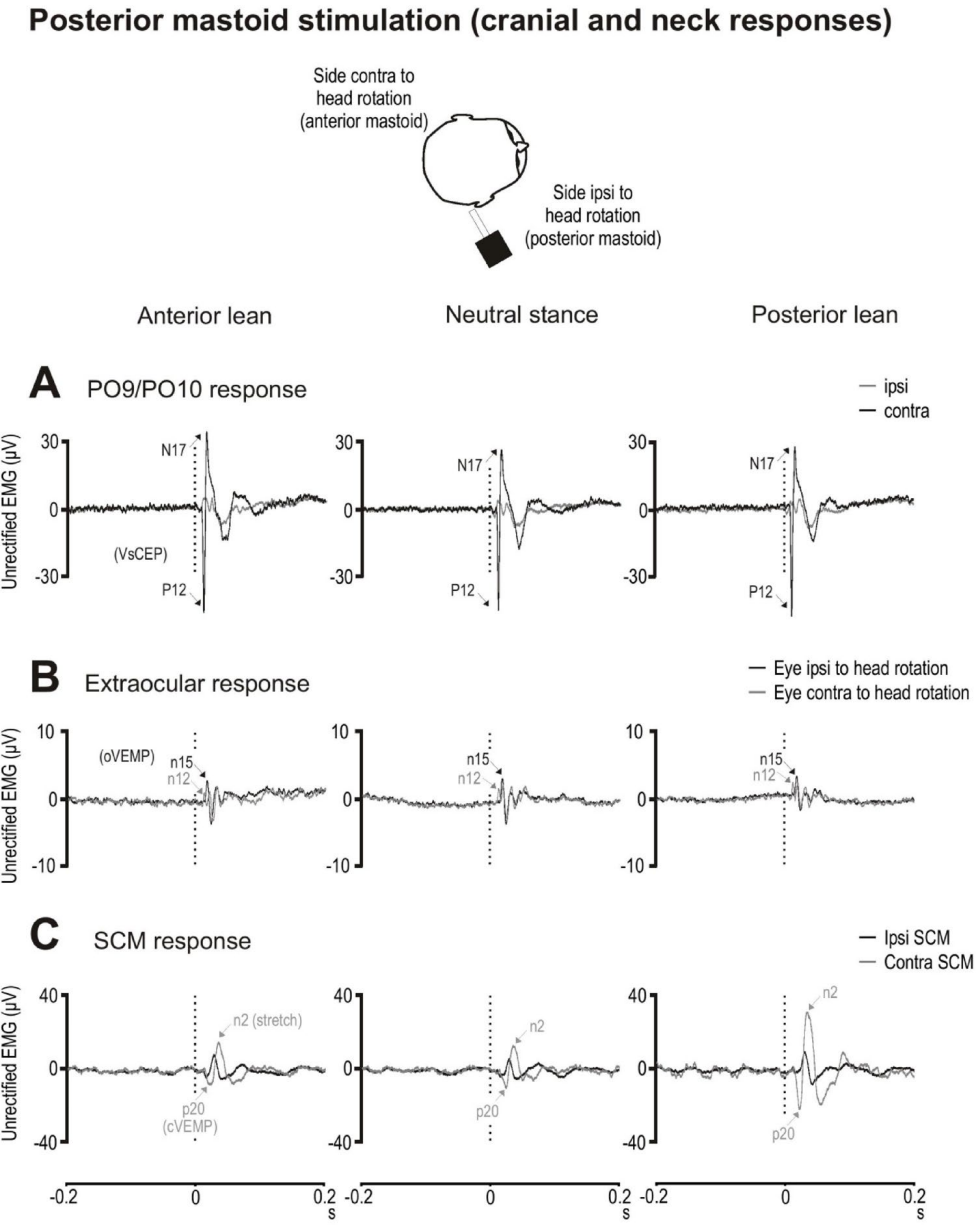
Grand mean evoked responses following posterior mastoid stimulation recorded from the PO9 (head right) PO10 (head left) electrodes (**A**), extraocular muscles (**B**) sternocleidomastoid (SCM) muscles (**C**) during anterior lean (left column), neutral stance (middle column) and posterior lean (right column) postural conditions

A large biphasic response at PO9/PO10 consisted of P12 and N17 peaks (Fig. 6A). Overall, there was no significant effect of posture on P12, N17 and peak-to-peak P12–N17 amplitudes or latencies (Table 4). P12, N17 and peak-to-peak P12–N17 amplitudes (F_(2,18)_ = 0.7 to 1.2, *p* = 0.338 to 0.498) and latencies (F_(2,18)_ = 1.5 & 0.3, *p* = 0.244 & 0.810) were unaffected across the three neutral stance conditions.

For oVEMPs, the n12 and p15 amplitudes were also not affected across postural conditions (F_(2,18)_ = 1.5 & 1.4, *p* = 0.256 & 0.270)(Fig. 6B). Peak latencies for the n12 potential showed a small change with posture (F_(2,18)_ = 4.5, *p* = 0.030, η_p_^2^ = 0.32), with anterior lean producing later potentials than neutral stance (13.0 vs. 11.5 ms, t_(9)_ = 2.7, *p* = 0.029, *d* = 0.9). Amplitudes and latencies for the n12 and p15 peaks were similar across the three neutral stance conditions (F_(2,18)_ = 0.1 to 1.9, *p* = 0.182 to 0.908).

SCM responses to posterior mastoid stimulation were modulated by tonic activity and consisted of cVEMP (p20) and stretch (n2) peaks. Both responses were larger on the side contralateral to head rotation and increased from anterior to posterior lean (Fig. 6C). Tonic activity in the contralateral SCM differed significantly across the lean conditions (F_(2,18)_ = 6.6, *p* = 0.024, η_p_^2^ = 0.42) with larger tonic levels during posterior lean compared to the neutral and anterior lean conditions (t_(9)_ = 2.5 & 2.7, *p* = 0.032 & 0.024, *d* = 0.8 & 0.9; Table 4). The p20 peak showed a trend towards being larger during posterior lean even after correcting for differences in tonic activity between postures (F_(2,18)_ = 4.0, *p* = 0.056). Posture also altered n2 (corrected ratio) amplitudes (F_(2,18)_ = 13.5, *p* = 0.001, η_p_^2^ = 0.60), with posterior lean producing significantly larger responses than neutral stance and anterior lean (t_(9)_ = 4.3 & 4.0, *p* = 0.002 & 0.003, *d* = 1.3 for both). There was a trend for n2 peak latencies to occur earlier during posterior lean (F_(2,18)_ = 3.4, *p* = 0.085).

#### Anterior mastoid stimulation

Anterior mastoid stimulation provides a posteriorly-directed transient resisted by TA activation or soleus inhibition, the converse of posterior mastoid stimulation. For TA (agonist), tonic activity was highest during posterior lean and for soleus (antagonist) during anterior lean.

**Fig. 7:**
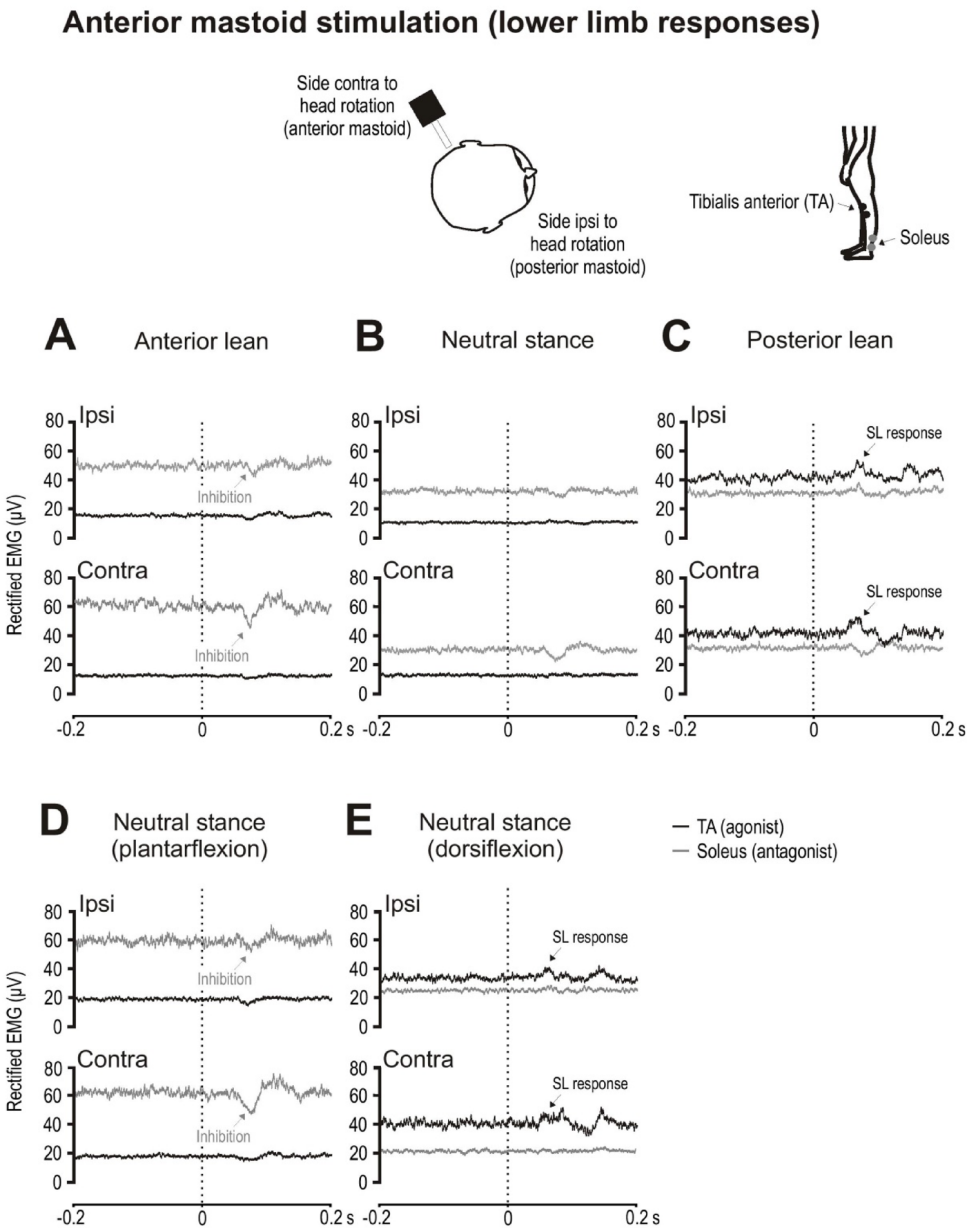
Grand mean EMG evoked responses to anterior mastoid stimulation during leaning and upright postures. Responses are shown ipsilateral and contralateral to the direction of head rotation. Posterior lean produced larger tonic levels in TA while anterior lean increased tonic levels in soleus, with both agonist (TA; black traces) and antagonist (soleus; grey traces) muscles producing SL responses of differing polarity (**A**–**C**). Responses were observed in the soleus muscles during neutral stance with plantarflexion (inhibitory, **D**) and in the TA muscles with dorsiflexion (**E**)

For TA, the excitatory SL response was largest when posture was most threatened (posterior lean with anterior mastoid stimulation; Fig. 7C). Neutral stance with dorsiflexion increased TA tonic levels (Fig. 7E) and closely matched the tonic levels recorded in TA during posterior lean (48.5 ± 32.5 µV vs. 37.1 ± 33.8 µV; t_(9)_ = 0.8, *p* = 0.433). With similar tonic activation levels for TA, there was a trend for SL responses to be larger during posterior lean compared to neutral stance with dorsiflexion (13 ± 7% vs. 7 ± 6%, t_(9)_ = 1.9, *p* = 0.087; Table 5).

For soleus, anterior lean produced inhibitory SL responses (Fig. 7A) with mean amplitudes of − 7 ± 9% (ipsilateral) and − 13 ± 5% (contralateral), with responses slightly larger on the contralateral side (t_(9)_ = 2.3, *p* = 0.048, *d* = 0.7). During neutral stance with plantarflexion (*p* = 0.540 for tonic EMG levels; Fig. 7D), mean amplitudes for the inhibitory SL responses were − 3 ± 9% (ipsilateral) and − 14 ± 5% (contralateral). There was no difference in the magnitudes of the inhibitory SL responses for the ipsilateral (t_(9)_ = 1.1, *p* = 0.294) nor for the contralateral soleus (t_(9)_ = 0.6, *p* = 0.571).

**Fig. 8:**
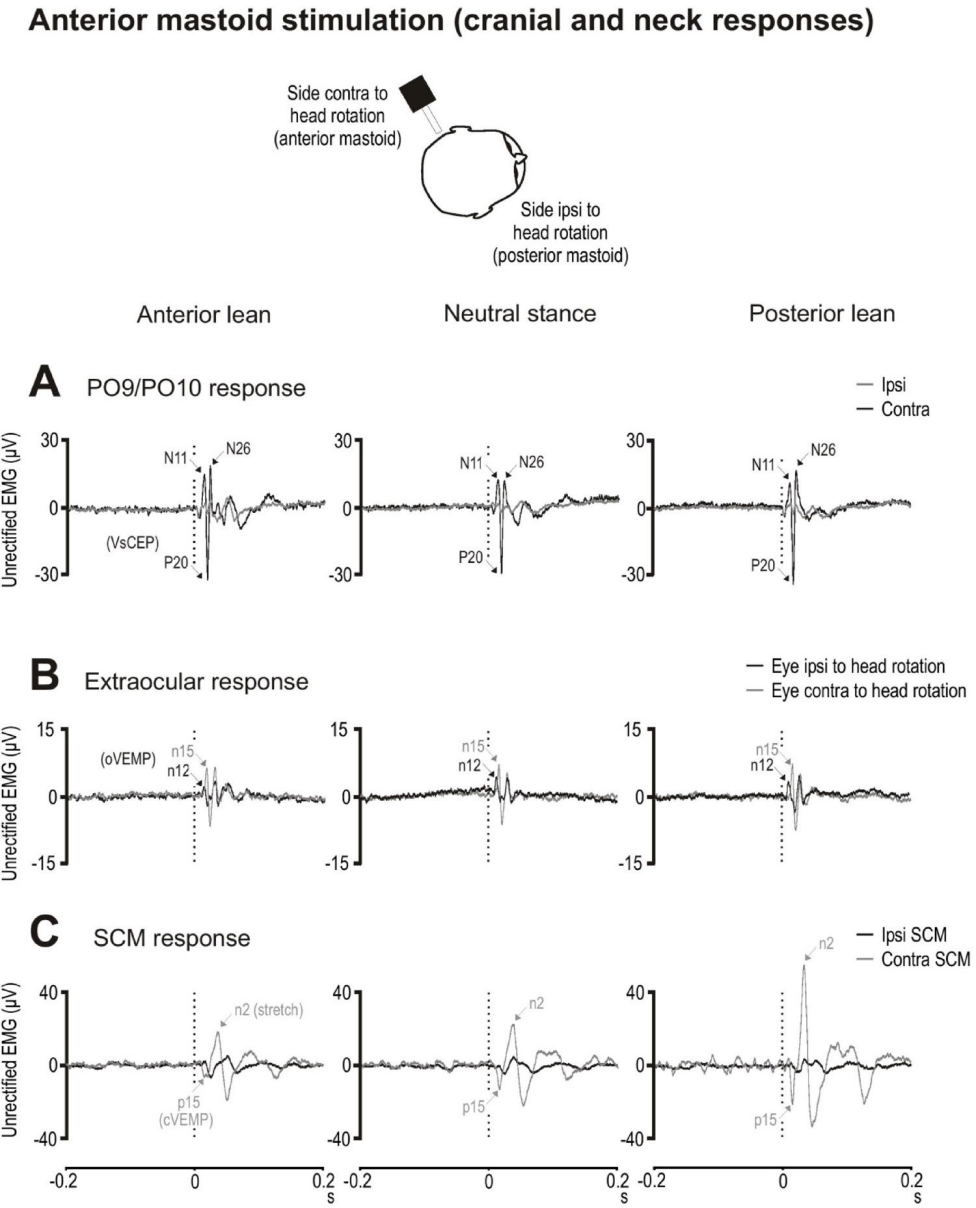
Grand mean evoked responses following anterior mastoid stimulation recorded from the PO9 (head right) and PO10 (head left) electrodes (**A**), extraocular muscles (**B**) and sternocleidomastoid (SCM) muscles (**C**) during anterior lean (left column), neutral stance (middle column) and posterior lean (right column) postural conditions

For the PO9/PO10 electrodes, larger responses were produced on the side contralateral to head rotation as previously reported (Govender et al. 2024a; Fig. 8A). However, N11, P20 and N26 peak amplitudes did not differ significantly across postures (Table 6). N11 peak latencies tended to be later during anterior lean (F_(2,18)_ = 4.2, *p* = 0.056), while for the N26 peak there was a trend for the response to peak later during neutral stance (F_(2,18)_ = 4.0, *p* = 0.062). The N11, P20 and N26 peaks were unaffected across the three neutral stance conditions (amplitude: F_(2,18)_ = 0.1 to 1.3, *p* = 0.303 to 0.919; latency: F_(2,18)_ = 0.1 to 1.2, *p* = 0.340 to 0.901).

For the extraocular electrodes, oVEMPs were produced bilaterally (Fig. 8B). Neither the n12 and n15 peak amplitudes nor latencies were affected across postural conditions (Table 6). The n12 and n15 peaks were also similar in amplitude (F_(2,18)_ = 0.1 & 0.8, *p* = 0.946 & 0.457) and latency (F_(2,18)_ = 0.1 & 0.5, *p* = 0.899 & 0.618) across the three neutral stance conditions.

For the SCM muscles, head rotation produced greater tonic activation on the side contralateral to head rotation and consisted of the initial p15 cVEMP peak and the later stretch (n2) response (Fig. 8C). Both the p15 and n2 peak amplitudes increased from anterior to posterior lean (Table 6) and both were increased with leaning posture even after rescaling for differences in tonic levels (F_(2,18)_ = 5.4 & 10.1, *p* = 0.021 & 0.002, η_p_^2^ = 0.38 & 0.53). Posterior lean produced enlarged p15 and n2 ratio amplitudes to a similar degree compared to the neutral and anterior lean conditions (p15: t_(9)_ = 2.3 & 3.0, *p* = 0.045 & 0.015, *d* = 0.7 & 1.0; n2: t_(9)_ = 3.4 & 3.6, *p* = 0.008 & 0.006, *d* = 1.1 & 1.2). Latencies for the n2 peak were also affected (F_(2,18)_ = 7.2, *p* = 0.009, η_p_^2^ = 0.45) with the response occurring earlier during posterior lean (t_(9)_ = 3.0 & 3.1, *p* = 0.013 & 0.012, *d* = 1.0 for both).

**Table 6:** Tonic EMG, SCM, PO9/PO10 electrode and extraocular responses to anterior mastoid stimulation during anterior, neutral and posterior lean conditions (*n* = 10)

Anterior mastoid stimulation				
	Anterior lean	Neutral stance	Posterior lean	*p* value
Tonic EMG (lower limb muscles)				
TA (µV)	13.8 ± 9.0	11.5 ± 6.7	48.5 ± 32.5	0.003**
Soleus (µV)	56.3 ± 42.7	32.2 ± 19.8	21.7 ± 5.4	0.008**
PO9/PO10 response (electrode contralateral to head rotation)				
N11 amp (µV)	18.8 ± 12.1	15.1 ± 7.3	13.0 ± 11.8	0.242
P20 amp (µV)	49.9 ± 38.3	46.1 ± 33.4	52.3 ± 53.5	0.859
N26 amp (µV)	36.3 ± 34.2	31.6 ± 32.7	33.0 ± 37.1	0.798
N11 latency (ms)	12.0 ± 0.8	11.0 ± 1.2	11.2 ± 1.0	0.056
P20 latency (ms)	16.7 ± 1.3	16.8 ± 1.5	16.5 ± 1.3	0.438
N26 latency (ms)	21.0 ± 1.3	22.1 ± 2.4	21.3 ± 1.3	0.062
Extraocular response				
n12 amp (µV)	3.3 ± 2.1	3.7 ± 3.0	3.3 ± 3.0	0.707
n15 amp (µV)	7.4 ± 4.5	6.8 ± 4.7	6.7 ± 4.9	0.663
n12 latency (ms)	12.1 ± 1.7	12.4 ± 1.8	12.4 ± 1.9	0.571
n15 latency (ms)	15.1 ± 0.8	15.5 ± 0.9	15.5 ± 0.9	0.116
SCM response (SCM contralateral to head rotation)				
Tonic levels (µV)	36.5 ± 17.5	39 ± 19.2	66.6 ± 37.2	0.025*
p15 peak amp (µV)	8.0 ± 10.2	14.2 ± 17.9	30 ± 29.4	0.024*
p15 peak ratio	0.2 ± 0.2	0.3 ± 0.3	0.4 ± 0.3	0.021*
n2 peak amp (µV)	23.3 ± 23.1	31.0 ± 26.9	84.0 ± 62.3	0.013*
n2 peak ratio	0.7 ± 0.7	0.8 ± 0.6	1.4 ± 0.9	0.002**
p15 latency (ms)	15.7 ± 1.4	15.0 ± 1.2	15.1 ± 0.9	0.415
n2 peak latency (ms)	40.8 ± 4.6	38.9 ± 3.6	35.4 ± 4.6	0.009**

Mean (SD), amp = amplitude, Ratio = raw amplitude divided by tonic activity. Greenhouse–Geisser corrected *P* values are given. * *p* < 0.05, ** *p* < 0.01

### Accelerometry

For C7 stimulation, the trunk accelerated forward with an initial peak of 0.11–0.13 mg at 7.9–8.4 ms across the postural conditions (Supplementary Fig. 1A). Head acceleration along the naso-occipital axis showed an initial backward movement (0.03–0.04 mg at 6.8–7.5 ms) followed by a larger forward acceleration (0.07–0.08 mg at 11.5–12.3 ms). Vertical acceleration was initially upward (0.06–0.07 mg at 17.4–18.6 ms), with a smaller lateral movement along the interaural axis (0.02–0.03 mg at 19.0–20.8 ms). Acceleration amplitudes and latencies did not differ across postures (F_(4,16)_ = 0.8–2.6, *p* = 0.137–0.552).

For sternal stimulation, the trunk accelerated backward with an initial peak of 0.07 − 0.10 mg at 5.8–7.0 ms across the postural conditions (Supplementary Fig. 1B). Initial head movements were backward (0.04 mg at 11.8–12.2 ms), downward (0.06–0.08 mg at 18.8–19.1 ms), and medial (0.02 mg at 17.5–18.3 ms). Posture had no effect on acceleration amplitude or latency (F_(4,16)_ = 0.2–2.3, *p* = 0.162–0.958).

For posterior mastoid stimulation, both the trunk (0.04–0.05 mg at 7.9–9.0 ms) and head (0.16–0.21 mg at 7.9–8.3 ms) accelerated forward with initial head movement in the downward (0.12–0.14 mg at 13.7–14.4 ms) and lateral directions (0.18–0.21 mg at 4.7–4.9 ms) (Supplementary Fig. 2A). Anterior mastoid stimulation produced backward trunk (0.03–0.04 mg at 7.5–7.8 ms) and head (0.18–0.22 mg at 8.1–8.7 ms) accelerations with initial head movement in the upward (0.05–0.08 mg at 7.2–8.4 ms) and lateral directions (0.08–0.11 mg at 4.7–5.4 ms) (Supplementary Fig. 2B). Overall, acceleration amplitude and latency were unaffected across postural conditions for posterior (F_(4,16)_ = 0.1–1.4, *p* = 0.776–0.307) and anterior (F_(4,16)_ = 0.6–3.3, *p* = 0.696 − 0.102) mastoid stimulus sites.

### Voluntary lean and CoP displacement

Voluntary lean from an upright neutral stance was 55.4 ± 22.8 mm anteriorly and 31.7 ± 13.7 mm posteriorly across stimuli. Axial stimulation produced peak CoP displacements similar to our earlier report (Govender et al. 2015). Peak CoP displacements were small and occurred well after the reflexes we measured here (Supplementary Fig. 3A, B). The range of mean latencies for peak CoP displacement was 128.5–155.9 ms (onset: 69.4–72.8 ms) for C7 stimulation and 119.2–132.5 ms (onset: 70.0–71.8 ms) for sternal stimulation. The peak CoP displacement for C7 stimulation was 0.9 ± 0.6 mm (anterior lean), 0.7 ± 0.5 mm (neutral stance), 0.5 ± 0.2 mm (posterior lean), 1.2 ± 0.9 mm (dorsiflexion) and 0.8 ± 0.7 mm (plantarflexion). For sternal stimulation, peak CoP displacement was − 0.4 ± 0.8 mm (anterior lean), − 0.4 ± 0.7 mm (neutral stance), − 1.4 ± 0.6 mm (posterior lean), − 1.1 ± 0.5 mm (dorsiflexion) and − 0.7 ± 0.5 mm (plantarflexion). CoP displacement changes were minimal with mastoid stimulation (Supplementary Fig. 3C, D).

## Discussion

Levels of tonic activation clearly have an effect on reflex size, often nearly linear (Colebatch et al. 1994; Rosengren 2015). Tonic levels of muscle activation are important determinants of reflex responses and thereby act as automatic gain control (Matthews 1986). While linear scaling by the tonic level of activation partly allows for this, the only way to remove it as a factor in determining reflex size is to match levels of activation. This we have done quite successfully, for both TA and soleus, allowing us to examine the effects of perceived postural instability separately from that of tonic activation. The perturbations themselves were unaltered by posture. We have confirmed a significant effect of instability on the response of soleus to postural disturbances evoked by non-vestibular (axial) stimulation. In the case of axial stimulation applied to the sternum, the responsible inputs are likely to be muscle afferents, including those from the SCM (Todd et al. 2022). The effect size was considerable for the reflex amplitude in soleus following C7 axial stimulation. The effects for TA were much less marked and these failed to reach significance following sternal stimulation. The selectivity for soleus may be due to postures with anterior lean, such as when reaching for objects, are much more common than those associated with posterior lean. Our findings are potentially applicable to larger perturbations because we have shown that the SL reflexes evoked by these small perturbations are significantly correlated with the initial postural responses evoked by larger, more directly threatening postural disturbances (Colebatch and Govender 2018).

Although important, the tonic level of activation does not determine the fundamental nature of the postural responses evoked by axial stimuli. This was shown during the increased soleus activity with sternal stimulation and the increased TA activity with C7 stimulation. Despite higher tonic levels of contraction, the initial effect remained appropriate for compensation for the perturbation i.e. inhibition of contraction (Figs. 1C, 3D). Although the displacement of the subject was minimal in response to the stimulus (see also Govender et al. 2015), directional specificity was retained. As noted above, muscle receptors in the SCM are likely to contribute to the response and muscle spindles show remarkable sensitivity with clear phasing in response to very small displacements (Matthews and Stein 1969). This observation also suggests that the effects of the perturbations are not simply determined by excitability at the spinal level and thus it is likely that distinct descending volleys are evoked by anterior and posterior axial perturbations.

Adkin et al. (2000) showed that postural sway reduced with increasing levels of perceived postural threat (using height above the surface). The mechanism of this effect was not clear but increased stiffness at the ankle (Carpenter et al. 1999) and greater voluntary control (Huffman et al. 2009) have both been proposed. Given their role in posture, vestibular reflexes have been natural targets for investigation. Horslen et al. (2014) showed increased vestibular coupling to reaction forces following stochastic vestibular stimulation when subjects stood on a high platform, but the relevance of vestibular reflexes has been questioned (Reynolds et al. 2015). Naranjo et al. (2015, 2016) found modest increases in vestibular evoked myogenic potentials (VEMPs) with subjects standing on increasing surface heights. We found increases in vestibular-dependent postural reflexes (evoked by mastoid stimulation) with leaning, for both soleus and TA although these were only statistical trends. We also showed some increase in cVEMPs, but this occurred in parallel with an increase in the vestibular-independent n2 reflex (Rosengren et al. 2009). In contrast, there was no amplitude change in the oVEMPs.

We recorded at Iz for axial stimulation and PO9/PO10 for vestibular stimulation, sites which lie over the cerebellum centrally and laterally. For both modes of stimulation, we have previously shown short latency evoked responses which appear to originate from the cerebellum (Govender et al. 2022; Todd et al. 2018, 2021). The vestibular-evoked P12–N17 VsCEPs at PO9/10 showed no change with differing postures but the P25, P50 responses at Iz evoked by axial stimulation in both directions, were larger with anterior lean. The latter, as argued above, are likely to depend upon muscle afferents. Todd et al. (2022) have reported that muscle afferents in the SCM evoke short latency responses from extraocular muscles and we found no change in the N26 extraocular potential with differing postures. In contrast to the N26 response, there was a large facilitation of the non-vestibular response in the SCM with leaning backwards. This too has been assumed to be of muscle afferent origin (Rosengren et al. 2009); Horslen et al. (2014) have argued for increased muscle spindle sensitivity with postural challenges. Against this, the increase on the cVEMP which also occurred was similar in magnitude to that for the n2. The n2 latency is considerably longer than would be expected for a segmental reflex, given that the efferent delay is around 6 ms (Brown et al. 1991), leading to an expected latency of around 12 ms. The SCM segmental reflex is also known to be weak (Keirstead and Rose 1988). Given the latency, it is possible this later reflex is mediated either transcortically as has been shown for limb muscles (Marsden et al. 1976) or via the brainstem, as we have shown for SCM afferent effects on leg muscles (Todd et al. 2022).

Overall we have shown a significant, selective increase for postural reflexes when subjects feel unstable. This applied in particular for the axially (C7) evoked postural responses in soleus but with no significant enhancement for the response in TA under similar circumstances. Vestibular-weighted responses showed weaker facilitation and our failure to show a significant effect may have been due to our sample size. The axial reflex is not dependent on intact vestibular function (Graus et al.^2013^) but vestibular afferents are likely to contribute (Govender et al. 2015). The short latency Iz potential (P25, P50) was also largest with anterior lean following axial stimulation, consistent with the selectively greater effect of lean on postural reflexes in soleus. This also provides evidence for brainstem involvement, particularly as we have reported that a similar short latency potential (P23, here P24), correlates with the size of the postural responses (Govender et al. 2024b). The mechanism of the facilitation is unlikely to be increased afferent volleys as the N21 and N26 external ocular responses (dependent upon muscle afferents) and oVEMPs (vestibular afferents) were unchanged. The basis of the enhancement we have demonstrated is thus most likely to be facilitation of the descending bulbospinal volleys evoked by the perturbations.

## Supplementary Information

Below is the link to the electronic supplementary material.

Supplementary Material 1

## Data Availability

Anonymised data will be made available through UNSWorks (unsworks.unsw.edu.au/home).
