# Supplementary material for: Selective facilitation of short latency postural reflexes by instability
Source: Exp Brain Res. 2025 Oct 4;243(11):220. doi: 10.1007/s00221-025-07168-8 (PMC12496292; doi:10.1007/s00221-025-07168-8)
Supplement: Supplementary file 1 — Supplementary Material 1 [file 221_2025_7168_MOESM1_ESM.docx]

**SUPPLEMENTARY FIGURES**

**
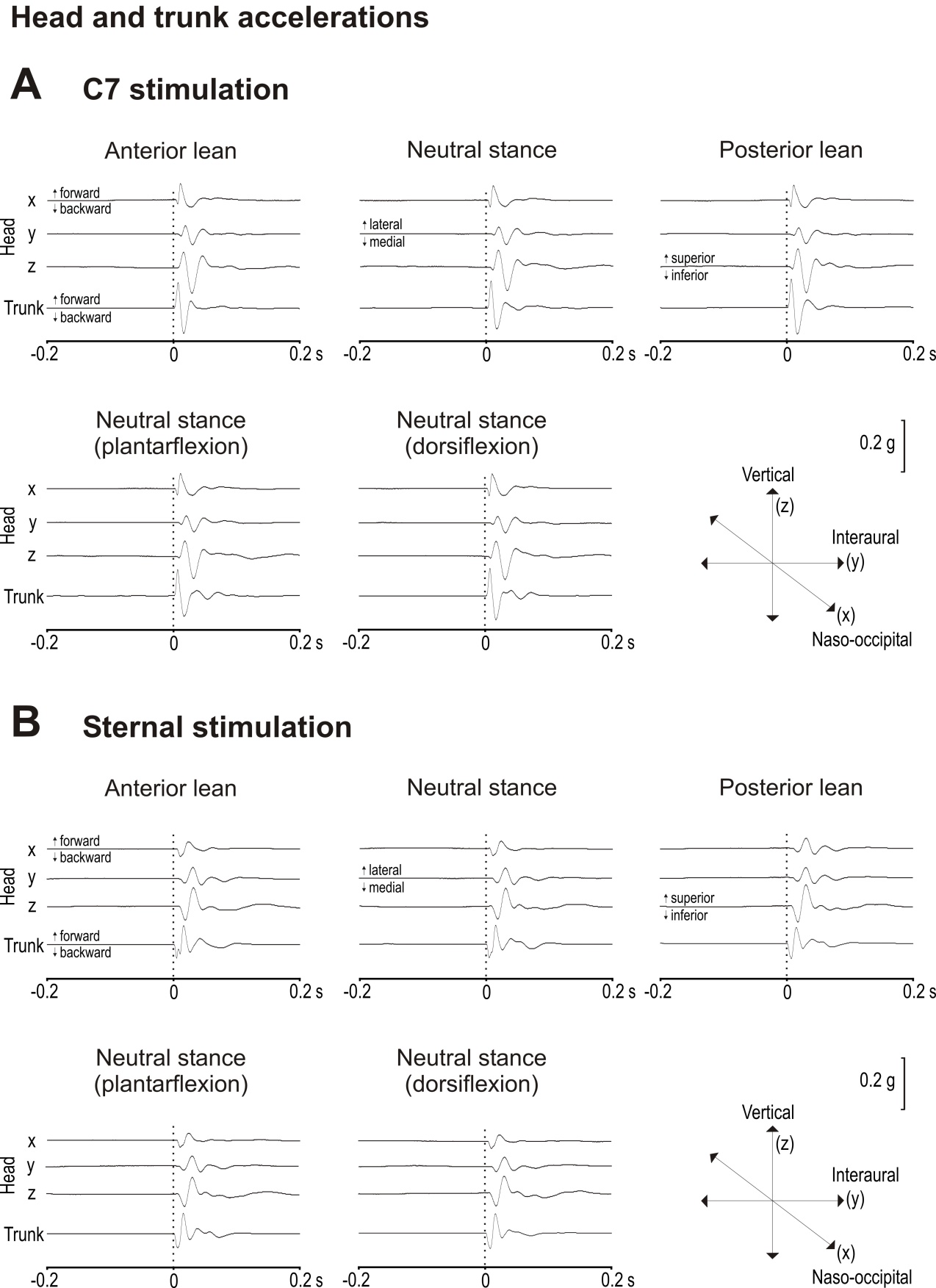
**

**Supplementary Fig. 1:** Grand mean acceleration traces from the head (x; naso-occipital axis, y; interaural and z; vertical axes) and trunk (naso-occipital) following C7 (A) and sternal (B) stimulation. Acceleration traces were similar across postural conditions for axial stimuli.

Stimulus given at time 0.

**
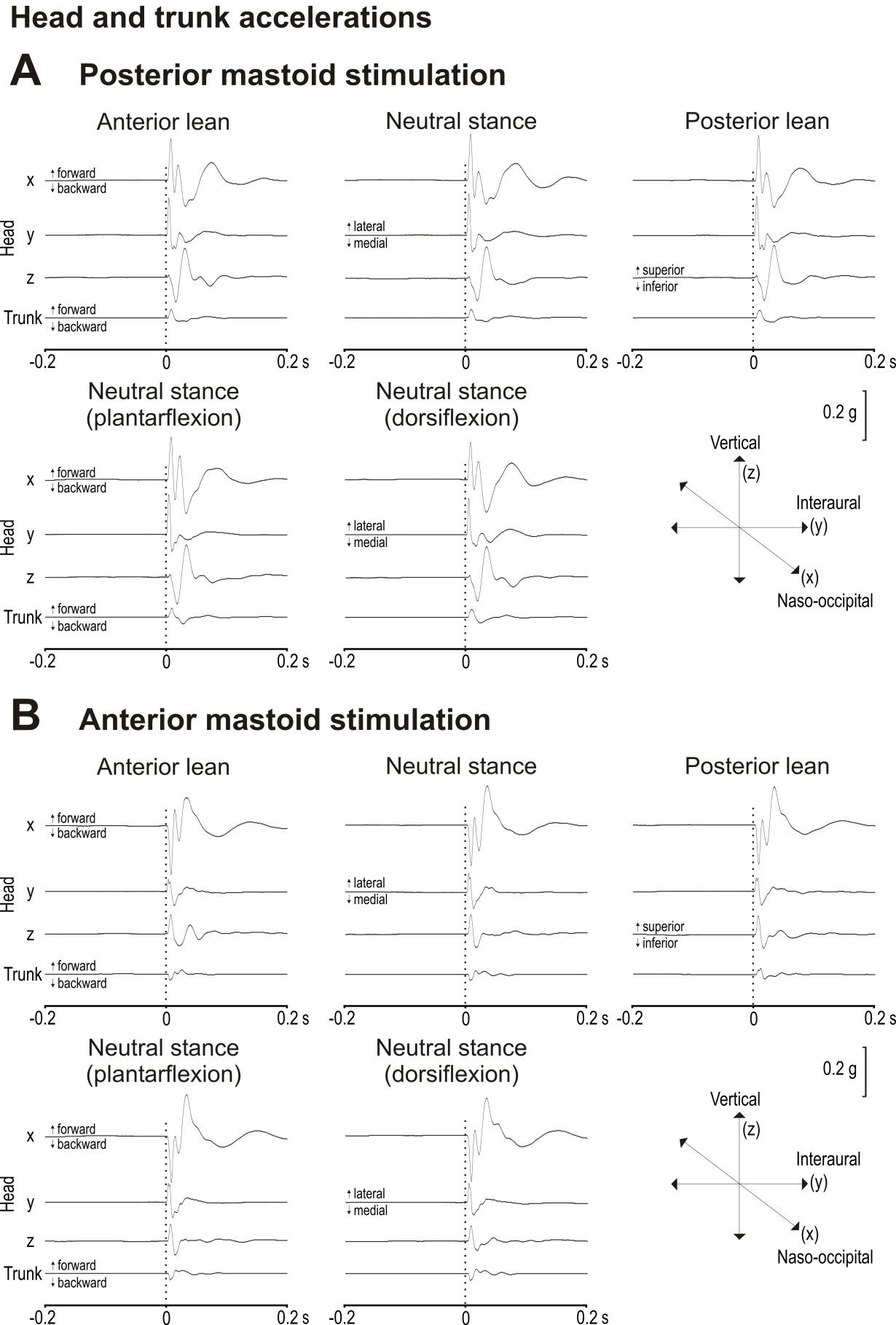
**

**Supplementary Fig. 2:** Grand mean acceleration traces from the head (x; naso-occipital axis, y; interaural and z; vertical axes) and trunk (naso-occipital) following posterior (A) and anterior (B) mastoid stimulation. Acceleration traces were similar across postural conditions for both directions of stimulation.

**
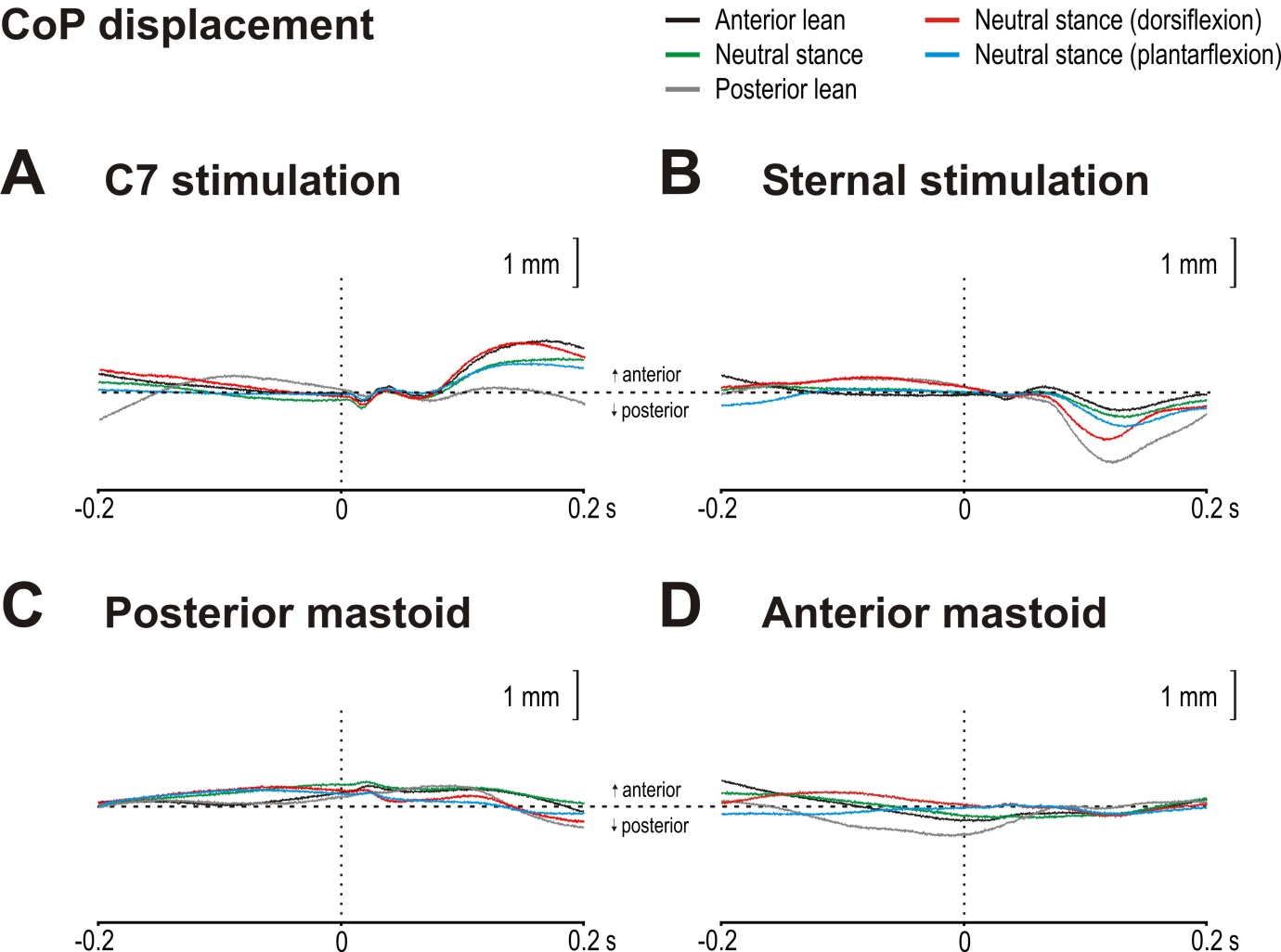
**

**Supplementary Fig. 3:** Centre of pressure (CoP) displacement following stimulation at (A) C7, (B) sternum, (C) posterior mastoid, and (D) anterior mastoid. Traces show mean CoP across the five postural conditions: anterior lean (black), posterior lean (grey), neutral stance (green), neutral stance with dorsiflexion (red), and neutral stance with plantarflexion (blue). C7 and sternal stimulation produced clear anterior and posterior CoP shifts, respectively, with little differences between conditions initially. CoP changes were smaller and showed similar changes following mastoid stimulation.
